# Best Practices for Data Sharing in Phylogenetic Research

**DOI:** 10.1371/currents.tol.bf01eff4a6b60ca4825c69293dc59645

**Published:** 2014-06-19

**Authors:** Karen Cranston, Luke J. Harmon, Maureen A. O'Leary, Curtis Lisle

**Affiliations:** National Evolutionary Synthesis Center, Duke University, Durham, North Carolina, USA; Department of Biological Sciences, University of Idaho, Moscow, Idaho, USA; Department of Anatomical Sciences, Stony Brook University, Stonybrook, New York, USA; KnowledgeVis, LLC, Maitland, Florida, USA

## Abstract

As phylogenetic data becomes increasingly available, along with associated data on species’ genomes, traits, and geographic distributions, the need to ensure data availability and reuse become more and more acute. In this paper, we provide ten “simple rules” that we view as best practices for data sharing in phylogenetic research. These rules will help lead towards a future phylogenetics where data can easily be archived, shared, reused, and repurposed across a wide variety of projects.

## Introduction

The amount of phylogenetic data has rapidly increased in its quality and availability over the past few decades. Additionally, phylogenetic matrices and trees are often based on and need to be linked to data on traits, geographic distribution, and genetic / genomic sequences. Despite the rapid growth in data generation, comparative data from published studies are too often unavailable, incomplete, or incompatible thereby greatly limiting reproducibility and expansion of existing studies 1 ^,^ 2. A greater focus on data integration and interoperability, even at the data collection phase of a project, allows for scalable, integrative analyses that combine data from multiple sources 3 ^,^ 4.

Modeled on other initiatives in science that have suggested practices to standardize and share data 5, here we discuss recommendations for data sharing that will allow the phylogenetics community to advance large scale research much more efficiently. “We” in this case, are members of the three NSF-funded AVAToL projects - Open Tree of Life, Arbor and Next-generation Phenomics, although this manuscript was heavily influenced by a period of public commenting 23 (see Acknowledgements).

We define phylogenetic data as the inputs, outputs and methodological details of a phylogenetic analysis. Current practices for publication of these data too often limit reusability. For example, molecular alignments are rarely preserved and made available; phenomic matrices do not always include full descriptions of characters and character states, character states do not follow standardized anatomical language, and despite the availability of inexpensive digital photography, phenomic homology is rarely documented with images; phylogenetic trees are too often published only as images instead of digital data files that we can read into another software package; published trees often contain ambiguities in interpretation due to inconsistencies in methods, software, and file formats; and tip labels (terminal taxa) in tree files and associated data matrices are not always meaningful or comprehensible to anyone outside the original study. Because of these (and other) problems, phylogenetic comparative studies can be difficult or impossible to replicate or expand upon. When these studies are replicated, inconsistencies can be impossible to track down. As a result, advancement in the field is greatly slowed by very preventable issues.

If you are producing any kind of phylogenetic data as part of your research publications, your data are much more likely to have an impact if they are easily understandable and reusable by others. In many cases, the raw data and output files from your analysis require additional curation than what has historically been expected in order to ensure that they are easily reusable. Here we propose a set of “best practices” for comparative biology. Our best practices include suggestions that will facilitate reproduction and reuse of the input and output data from phylogenetic analyses. We encourage practices that enable automated curation and access to phylogenetic comparative data. We also emphasize the importance of file validators, curation tools, and software that outputs standardized and reusable data.

The best general solution is to make your data available in a public repository and in a format that has existing parsers (computer programs that can import the file) and validation tools (computer programs that determine if the file has valid formatting and confirm that the data in the file can be easily extracted). A variety of parsing tools exist for phylogenetic packages such as Mesquite 6, PAUP 7, TNT 8, MorphoBank 3, TreeBase 9 and CIPRES 10 . Going a step further, the newly emerging NeXML 11 and PhyloXML 12 formats are examples of file types that have a published digital schema (format) that allows for validation by a computer. These new formats represent the future of standardization. Widespread implementation of such formats will eliminate much of the curation work that now wastes the time of many scientists. Although many widely used programs do not currently read and write these new XML formats, the field is moving in a direction to standardize communication and archivation using XML. A compromise that works at present is to publish all of the data in plain text files with documentation that describes the file contents, allowing for downstream use after some manual curation (Nexus or TNT files, for example, are both plain text format). Neglecting to publish the data files at all, or requiring extensive reading of the manuscript in order to make sense of the data, substantially reduced the interpretability, reusability and, ultimately, the value of your results. The following rules give specific examples of how to make your phylogenetic data understandable and how to increase its impact. Appendix 1 lists the predominant file formats in phylogenetics and the benefits and downsides of each form.

The following sections list ten simple rules for publishing and sharing phylogenetic data.

## Rule 1: Manage your data as if sharing matters, right from the start

In the initial stages of your project, think about what you are going to share and in what form. Agree with your co-authors on what your data legacy will be, how soon data will be shared, and how the data will be licensed. Do this by asking “What is needed to reproduce our results?” Keep track of which versions of the data are actually used in the published analysis. Assembling and analyzing your data while being cognizant of data sharing rules (e.g., writing taxon names in full) will, in the long run, save curation time in the end. Ideally, manage your data package using tools such as github that store old versions and allow you to keep notes (e.g., descriptions of changes) separate from the document. Resources such as MorphoBank support collaboration during the data collection phase, and allow you to release your private data set to a public archive upon publication.

## Rule 2: Publish your data (and not just pictures of your data!)

The word “publish” has taken on expanded meaning in the digital age. Previously, the most familiar connotation of the word “publish” for scientists was to have a paper accepted and presented by a peer-reviewed academic journal. In the age of data sharing and digital re-use policies, “publishing your data” means to deposit it in a digital archive that makes the data broadly available to the community. A surprising number of phylogenetic studies publish matrices or trees only as a figure in a printed journal article, a format that makes reuse at best challenging and often impossible. Publish character matrices, genetic sequence alignments, and your phylogenetic trees as one or more digital files in a data repository such as TreeBASE, Dryad, MorphoBank or a DataOne member node. Include the kind of data that a colleague would need in order to verify your results, build on your analysis, or include your data in a meta-analysis. If a standard format for your type of data is available (e.g., standard alignment formats for DNA), use it (see Appendix 1). In all cases, use plain text formats (for example, .txt or .csv files) rather than proprietary and non machine-readable formats such as Microsoft Office documents or PDFs.

## Rule 3: CC0 maximizes ease of data reuse

Input data to a phylogenetic analysis are scientific facts, not subject to copyright, and performing a computational analysis on scientific facts does not constitute the type of creative expression subject to copyright law 24 . Creative expression, such as images, can be subject to copyright. Newer databases and initiatives, such as as Dryad and Open Tree of Life, require that uploaded data be marked with an explicit CC0 waiver, which means that the author legally waives any claim to copyright. We recommend using the CC0 waiver for phylogenetic data. The Dryad data repository points out three key benefits to CC0:

1. Interoperability: CC0 is human and machine-readable, allowing people and services to easily determine terms of use.

1. Universality: CC0 is widely recognized, covering all data and all countries.
2. Simplicity: eliminates the need for humans to make, and respond to, individual data requests, allowing scientists to spend their time doing science.

There are mature resources that contain phylogenetic data (for example, Genbank, TreeBASE or Morphobank) and have not placed copyright licenses or waivers of any kind on gene sequences, trees, matrices and other associated data. It should be understood by the phylogenetics community that those databases contain content to be shared and the very reason those databases came into existence is to share their content to further scientific research. However, there may be legal issues (beyond the scope of this paper) that come up when combining data that does not have explicit licensing, waivers or terms of use.

## Rule 4: Include a README file in your data package

A README file is a plain text file that describes the contents of your data package, generally listing each file in the package with a description of its contents. Dryad has examples of data packages well-documented using README files 13 ^,^ 14 ^,^ 15 . Such files can also be stored in the Documents folder on MorphoBank 16 where they can be shared during peer review. When writing your README file for your data package, note that meaningful file names can simplify description of the package and subsequent understanding by reviewers or users.

## Rule 5: Provide meaningful labels for taxa

The labels that you use for terminal taxa (“tips”) in your tree should be meaningful to someone not familiar with your study. Avoid using lab codes, abbreviations or common names. Use full taxon names (not abbreviations like “*C. elegans*”) or use identifiers from an online database (e.g., NCBI, Paleobiology Database). For studies where Linnaean taxon names are not appropriate or not sufficient (for example, studies that contain multiple individuals per species, studies containing unnamed taxa or microbial studies where strain is important), use a consistent format for names that includes additional information such as specimen numbers, strains or accession number. Increasingly, online databases used in phylogenetic work contain information about taxa, strains, specimens, etc (GenBank, PaleoDB, GBIF, SILVA) and we encourage linking taxon labels to online resources to help disambiguate and cross-reference other data. XML file formats allow you to do this within the same file. Alternately, provide a mapping file that lists the taxon names in a phylogeny and the corresponding entity in an online resource. For phenomic data, MorphoBank provides a single data management and publishing platform that allows dynamic linking of taxon labels to online resources.

## Rule 6: Use the same taxon labels across different data elements or files

The taxon names in your phylogenetic tree should match those in the alignment, character matrix, and other data elements, whether they are in the same file or different files. In cases where you must use different taxon labels across your study, include explicit information about how one set of labels maps to another. The best solution is to use a file format that allows for this mapping within the file (e.g. NeXML, PhyloML, STK-XML 17 , or a TaxaAssociation block in a Mesquite-style NEXUS file; see Appendix 1). An alternate is to include the mapping information in a separate text file.

## Rule 7: Provide all of the data and scripts needed to replicate your analyses

Rather than simply describing your analysis in the Methods section of a publication, also deposit in a public data repository any input files and/or scripts that you used to produce the phylogeny. For example, publishing a MrBayes block, BEAST XML or R script ensures that all of the inference parameters and default values are clearly stated. If you ran the analysis using command-line options rather than putting the commands in a file, for example with RaXML 18 or Muscle 19, include the full command that you used in a README file. Include in the data package all alignment files needed to rerun your analyses. If trees are reconstructed from multiple alignments, each alignment should be made available. Which alignment corresponds to which published tree should be explicit. In the case of concatenated alignments, provide metadata about the beginning and end of each gene (e.g., in NEXUS, a SETS block with CHARSET or CHARPARTITION commands).

## Rule 8: Provide information about the tree as well as the tree itself

A tree topology is difficult to interpret and of minimal use without associated information. The Minimum Information About a Phylogenetic Analysis (MIAPA) checklist 20 provides a reference list of useful tree annotations. This includes information such as branch lengths, branch length units, support values, and rooting. When publishing your data, ensure that the data package contains sufficient information for comprehension and reusability, either in the phylogeny files themselves or in an associated README file. Rooting is a particularly problematic annotation, as changes in rooting can greatly impact phylogenetic statements, and the tree file output from software is often not rooted in the same way as the figures in the paper. If a tree is rooted in the paper, publish your data file with the tree rooted in the same way. It is best to indicate rooting through the orientation of the tree itself (for example, in a Newick string, the outermost parentheses indicate the root), rather than annotating the root separately from the tree structure using software-specific commands (for example, using the unrooted [&U] annotation or stating an outgroup that differs from the rooting implied by the tree structure).

## Rule 9: Provide complete and easily reusable information about characters, character states, and the specimens from which they are derived

For phenomic matrices (and combined matrices by extension), if you need to add a new taxon to an existing matrix or a new set of characters to an existing matrix, having easily accessible information about characters and states make the original data much easier to expand upon.

Phenomic data are far more reusable if the character names and states are embedded in the same file as the matrix itself, if the format being used permits this practice (e.g., Nexus, TNT). An impediment to reuse is the storage of a matrix without such information and separate pdf or Microsoft Word file with the character names and states. Archive any ordering (weighting) rules used in the analyses of the character data, either in that file, so that they are readily readable by tree search programs or at the very least in a separate file. Whenever practical, include journal citation/source of character state information to each character. That way, errors or conflicts can be easily tracked down to the original source and corrected. As noted in the Rule 1, it is much easier to organize this information if a plan to do so is developed at the start of the project, not at the end.

## Rule 10: Educate yourself on data management practices in your field

Technology is changing and standards for data archiving will evolve over time. Formats and databases in use today (Appendix 1) will change every year. Your data may pose a special challenge for sharing that only you can address. Improve your expertise by reading what has been written on data archiving in your field and in related fields and by consulting with colleagues or the help documentation of different software packages. This list provides a good starting point but is not meant to be comprehensive:

- The Joint Data Archiving Policy, adopted by dozens of evolution-related journals: http://datadryad.org/pages/jdap
- Data archiving in ecology and evolution: best practices 21

- Sharing and re-use of phylogenetic trees (and associated data) to facilitate synthesis 2
- Open Data and the Social Contract for Scientific Publishing 22
- The Panton Principles: http://pantonprinciples.org/

- Sharing Publication-Related Data and Materials: Responsibilities of Authorship in the Life Sciences (National Research Council (US), 2003): http://www.nap.edu/catalog.php?record_id=10613
- Data One best practices guide: http://www.dataone.org/all-best-practices

## The future of data sharing in phylogenetics

Our vision in this paper is a future where phylogenetic data are seamlessly archived, shared, reused, and repurposed across a wide variety of projects. This can be facilitated by a few changes in “standard practices” in phylogenetics, many of which are already partially underway. For example, analyses will likely move from desktops onto the web; data file formats are already moving from structured text, unique to each application, to standardized and validatable files; controlled vocabularies and ontologies are providing consistent, machine-readable language across projects; and character data is increasingly tied to databases of ontological information and image files that fully describe and document those characters. This transition can be aided by software developers, who can write software that uses interoperable data formats; by journal editors and reviewers who develop and enforce data sharing requirements; and by end-users, who can embrace the culture of data sharing and reuse that promises to transform comparative biology.

## Appendix 1

### File formats for phylogenetic data

Download PDF
